# A multicenter prospective phase III clinical randomized study of simultaneous integrated boost intensity-modulated radiotherapy with or without concurrent chemotherapy in patients with esophageal cancer: 3JECROG P-02 study protocol

**DOI:** 10.1186/s12885-020-07387-y

**Published:** 2020-09-22

**Authors:** Lin-rui Gao, Xin Wang, Weiming Han, Wei Deng, Chen Li, Xiaomin Wang, Yidian Zhao, Wenjie Ni, Xiao Chang, Zongmei Zhou, Lei Deng, Wenqing Wang, Wenyang Liu, Jun Liang, Tao Zhang, Nan Bi, Jianyang Wang, Yirui Zhai, Qinfu Feng, Jima Lv, Ling Li, Zefen Xiao

**Affiliations:** 1 Department of Radiation Oncology, National Cancer Center/National Clinical Research Center for Cancer/Cancer Hospital, Chinese Academy of Medical Sciences and Peking Union Medical College, Beijing, 100021 China; 2 Department 4th of Radiation Oncology, Anyang Cancer Hospital, Anyang, 455000 China; 3 Department of Oncology, Affiliated Tengzhou Central People’s Hospital of Jining Medical University, Jining Medical University, Tengzhou, 277599 China

**Keywords:** Esophageal cancer, Concurrent chemoradiotherapy, Definitive chemoradiotherapy/radiotherapy, Consolidated chemotherapy, Simultaneous integrated boost, Intensity-modulated radiotherapy, Randomized controlled trial

## Abstract

**Background:**

Since the development of three-dimensional conformal radiotherapy and intensity-modulated radiotherapy (IMRT), no prospective study has investigated whether concurrent chemoradiotherapy (SIB-IMRT with 60 Gy) remains superior to radiotherapy (SIB-IMRT) alone for unresectable esophageal cancer (EC). Furthermore, the optimal therapeutic regimen for patients who cannot tolerate concurrent chemoradiotherapy is unclear. We recently completed a phase I/II radiation dose-escalation trial using simultaneous integrated boost (SIB), elective nodal irradiation, and concurrent chemotherapy for unresectable EC. We now intend to conduct a prospective, phase III, randomized study of SIB-IMRT with or without concurrent chemotherapy. We aim to find a safe, practical, and effective therapeutic regimen to replace the conventional segmentation (1.8–2.0 Gy) treatment mode (radiotherapy ± chemotherapy) for unresectable EC.

**Methods:**

This two-arm, open, randomized, multicenter, phase III trial will recruit esophageal squamous cell carcinoma patients (stage IIA–IVB [UICC 2002]; IVB only with metastasis to the supraclavicular or celiac lymph nodes). In all, 164 patients will be randomized using a 1:1 allocation ratio, and stratified by study site and disease stage, especially the extent of lymph node metastasis. Patients in the SIB arm will receive definitive SIB radiotherapy (95% planning target volume/planning gross tumor volume, 50.4 Gy/59.92 Gy/28 f, equivalent dose in 2-Gy fractions = 60.62 Gy). Patients in the SIB + concurrent chemotherapy arm will receive definitive SIB radiotherapy with weekly paclitaxel and a platinum-based drug (5–6 weeks). Four cycles of consolidated chemoradiotherapy will also be recommended. The primary objective is to compare the 1-year, 2-year, and 3-year overall survival of the SIB + chemotherapy group and SIB groups. Secondary objectives include progression-free survival, local recurrence-free rate, completion rate, and adverse events. Detailed radiotherapy protocol and quality-assurance procedures have been incorporated into this trial.

**Discussion:**

In unresectable, locally advanced EC, a safe and effective total radiotherapy dose and reasonable segmentation doses are required for the clinical application of SIB-IMRT + two-drug chemotherapy. Whether this protocol will replace the standard treatment regimen will be prospectively investigated. The effects of SIB-IMRT in patients with poor physical condition who cannot tolerate definitive chemoradiotherapy will also be investigated.

**Trial registration:**

clinicaltrials.gov (NCT03308552, November 1, 2017).

## Background

The 2018 GLOBOCAN data estimated that approximately 572,000 people were newly diagnosed with EC in 2018, and that almost 509,000 people died of these cancers in the same year, making EC the seventh most common cancer and the sixth most common cause of cancer-related deaths [1]. In China, EC and esophagogastric junction cancer (EGJC) are the fouth most common types of cancer [2]; these malignancies always have a poor prognosis and respond poorly to treatment.

For patients with unresectable ECs (including patients with locally advanced EC or EGJC as well as patients who cannot undergo or refuse surgery), concurrent chemoradiotherapy is the standard treatment, and the recommended radiotherapy dose is 50.4 Gy based on the Radiation Therapy Oncology Group (RTOG) 85–01 [3, 4] and RTOG 94–05 trials [5]. However, these treatment and dose recommendations are currently considered controversial because of the following reasons. First, the randomized controlled trial part of the RTOG 85–01 study found that the 5-year overall survival (OS) rate after combined chemoradiotherapy was 26% compared with 0% following two-dimensional radiotherapy (2DRT) alone, which differs from the data reported in China [6, 7]. Over the past few decades, the 5-year OS rates after 2DRT with doses of 60–70 Gy have been reported to vary from 8.4 to 14.6% [6–8]. Second, the follow-up evaluation of the RTOG 85–01 study showed that disease persistence and locoregional recurrence were common modes of treatment failure, especially in the primary tumor region [4]. While it was lower in group who received combined therapy. Therefore, increasing the local radiotherapy dose to the primary tumor might be required to improve local control [9]. However, as reported in the RTOG 94–05 study, patients receiving high-dose radiotherapy (64.8 Gy) showed no improvement in terms of OS or local control, as compared with patients receiving low-dose radiotherapy (50.4 Gy). Thus, the optimal radiation dose remains to be determined. Finally, three-dimensional conformal radiotherapy (3DCRT) for unresectable EC yields 5-year OS rates of 34–45.6% [10–13], which is an improvement over the rates reported in the RTOG 85–01 and 94–05 studies. Moreover, radiotherapy (median dose, 60 Gy) with or without concurrent chemotherapy yields 5-year OS rates of 34.7 and 27.7%, respectively [14]. These results do not show a large difference in 5-year OS between radiotherapy with concurrent chemotherapy and radiotherapy alone, unlike the findings reported in the RTOG 85–01 study (27.7% vs. 0%, respectively). Although it was a retrospective study, it can also indicate that radiotherapy is the mainstay of treatment for EC, especially for patients who cannot tolerate concurrent chemotherapy. However, no prospective research study has been conducted to identify reasonable and effective doses of radiotherapy for EC.

The incidence of lymph node metastasis in EC is high, and the rate of early lymph node metastasis (i.e., in stage T1b) is 16.6–22.5% [15–17]; thus, preventive radiotherapy to the lymph nodes is essential. The simultaneous integrated boost (SIB) technique provides a suitable and heterogeneous dose distribution over a single radiation field. This technique is generally used to administer a high dose of irradiation to the tumor without significantly increasing the irradiation exposure of the organs at risk (OAR). However, as the esophagus has a lumen, administering a reasonable total dose of radiotherapy in multiple fractions is the basis of therapy. To evaluate this topic, we recently completed a phase I/II study of SIB intensity-modulated radiotherapy (IMRT) + two-drug chemotherapy for EC. We now intend to conduct a prospective, multicenter phase III clinical trial to determine whether SIB-IMRT with concurrent chemotherapy is sufficiently safe and effective to replace the standard treatment mode of conventional segmented radiotherapy (1.8–2.0 Gy) and concurrent chemotherapy. This study additionally aims to determine if SIB-IMRT alone is a suitable secondary treatment option for EC patients who cannot tolerate chemotherapy.

## Methods

### Study design and objectives

**Fig. 1:**
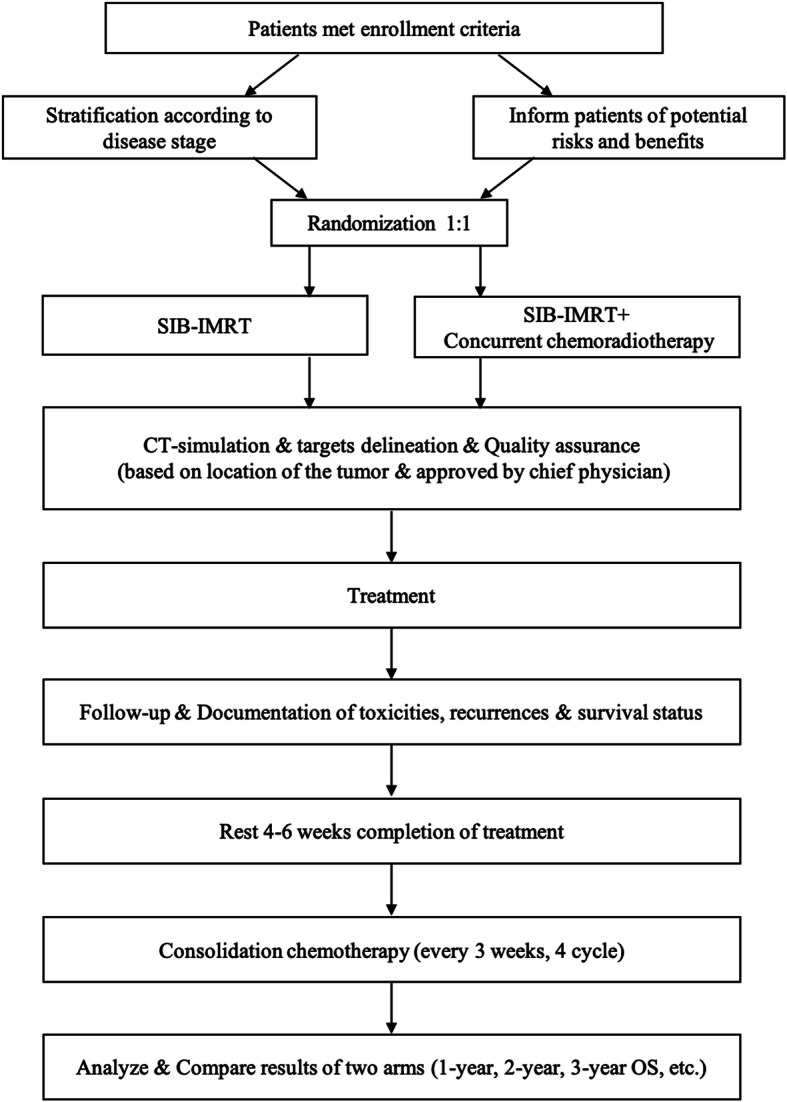
Flow chart of the 3JECROG P-02 trial

This study is an open label, randomized, comparative, multicenter study. The SIB technique will be used in this study, with the following dose regimen: 50.4 Gy/1.8 Gy/28 f to the planning target volume (PTV) and 59.92 Gy/2.14 Gy/28 f to the planning gross tumor volume (PGTV). Paclitaxel + nedaplatin will both be administered concurrent with radiotherapy. We randomly assigned (1:1) eligible patients, stratified by disease stage and tumor site, to one of four treatment groups: SIB + concurrent chemotherapy group or the SIB alone group. A flow chart giving an overview of the study design is shown in Fig. 1.

The coprimary objectives of this trial is to compare the 1-year, 2-year, and 3-year OS rates of the SIB + chemotherapy group and the SIB alone group. The secondary objectives consist of similar comparisons of the progression-free survival rate, local recurrence-free survival rate, treatment completion rate, and rate of adverse events. Patient recruitment for this study was started on September 1, 2017, and the duration of enrollment will be approximately 5 years.

### Patient selection

In this randomized phase III study, we recruited patients aged less than 70 years with histocytologically proven stage T2–4 N0–1 M1a (UICC 2002 [18]; stage IVB only with metastasis to the supraclavicular or celiac lymph nodes) unresectable esophagus squamous cell carcinoma (ESCC) of the are eligible for recruitment, no previous treatment before enrollment. Laboratory investigation requirements included the following: leukocytes ≥4.0 × 10^9^/L, neutrophils ≥3.5 × 10^9^/L, granulocytes ≥1.5 × 10^9^/L, platelets ≥100 × 10^9^/L, blood urea nitrogen ≤1.0 × upper normal limit (UNL), creatinine ≤1.0 × UNL, alanine aminotransferase/aspartate aminotransferase ≤1.5 × UNL, alkaline phosphatase ≤1.5 × UNL, and total bilirubin ≤ UNL. The general condition of the enrolled patients must also be acceptable: Karnofsky performance status score ≥ 70 or Eastern Cooperative Oncology Group performance status score ≤ 1, and Charlson Comorbidity Index score ≤ 3.

The exclusion criteria include age ≥ 70 years or < 18 years, prior chemotherapy or radiotherapy, pregnancy or lactation, known drug allergy, refusal to provide informed consent, insufficient hepatorenal function, abnormalities on routine blood examination (as defined above), severe cardiovascular diseases, diabetes with uncontrolled blood sugar level, mental disorders, uncontrolled severe infection, and active ulceration requiring intervention.

The elimination criteria include the following: (1) assigned patients did not match the study requirements, and (2) patients whose treatment was not performed as planned, those who developed unacceptable toxicity reactions, or those who withdrew from the study on their own accord. The study termination criteria are as follows: (1) disease progression during treatment, (2) other diseases that significantly affect the general condition of the patients and necessitate cessation of treatment, (3) unacceptable treatment toxicity, and (4) voluntary withdrawal from the trial at any time, according to the patient’s wishes.

### Radiotherapy

After completing the pretreatment examination, the following procedures will be performed: enhanced computed tomography (CT) for positioning and outlining the target area, determining the dose to be prescribed according to the modified radiotherapy plan, and submitting it to the physician to formulate the radiotherapy plan. Once the chief physician approves the plan, radiotherapy can be started. Cone beam CT-guided radiotherapy will be performed at least three times in the first week of radiotherapy and once a week thereafter.

The gross tumor volume (GTV-T) is defined as the encompasses the primary tumor, and is determined using all available resources {physical examination, upper gastrointestinal contrast, endoscopy, endoscopic ultrasonography [EUS], neck/thoracic/upper abdominal enhanced CT/MRI, positron-emission tomography [PET]-CT (if necessary), etc.}.

Lymph nodes diagnosed as metastatic or highly suspected as metastatic depending on the use of the physical examination and imaging tests (ultrasonography, CT, PET-CT, EUS, etc.) define as the metastatic regional nodes (GTV-N).

**Fig. 2:**
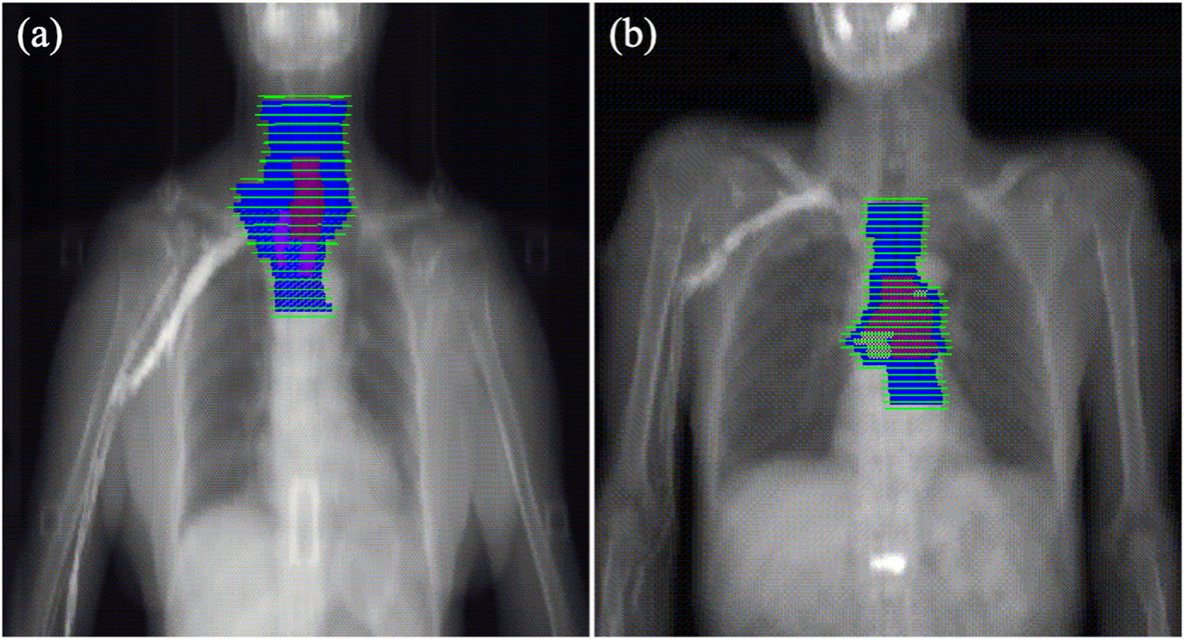
Target contouring of (**a**) the cervical esophagus and (**b**) the middle thoracic esophagus (Mt). The red area indicates the gross tumor volume (GTV-T); the grey area, the gross tumor volume for lymph nodes (GTV-N); the blue area, the planning gross tumor volume (PGTV); and the green area, the planning target volume (PTV)

**Fig. 3:**
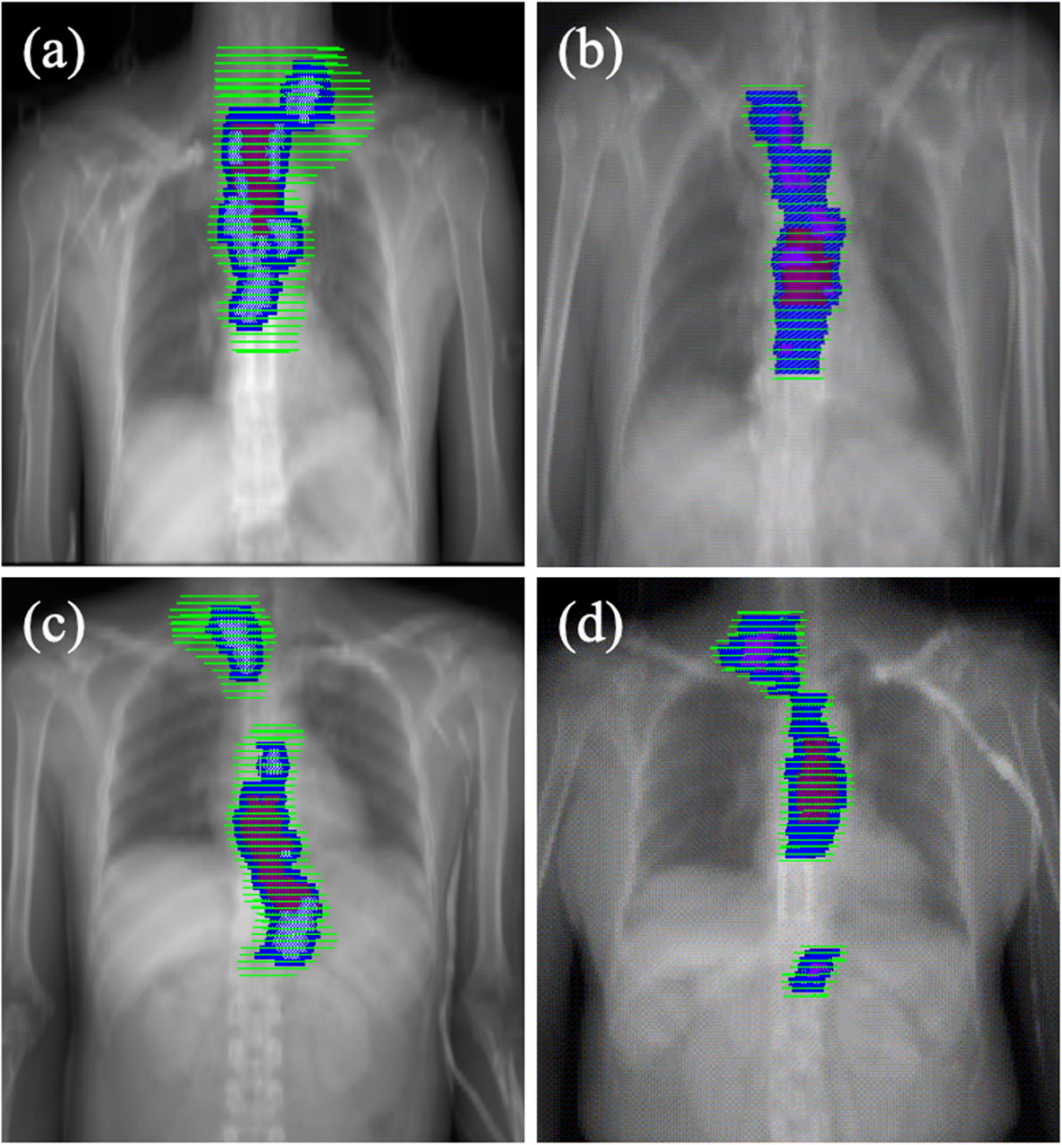
Target contouring for (**a, b**) elective nodal irradiation (ENI) and (**c, d**) involved-field irradiation (IFI). The red area indicates the gross tumor volume (GTV-T); the grey area, the gross tumor volume for lymph nodes (GTV-N); the blue area, the planning gross tumor volume (PGTV); and the green area, the planning target volume (PTV)

According to the clinical stage of the primary tumor and metastatic lymph nodes, the contouring of the clinical target volume (CTV) will be divided into two parts: elective nodal irradiation (ENI) and involved-field irradiation (IFI). ENI will include prophylactic irradiation of the draining lymph nodes. In such cases, the CTV is defined as the GTV with a 3.0–5.0 cm craniocaudal margin, a 0.6–0.8 cm lateral margin, and the corresponding draining lymph node area. For ECs with extensive lymphatic metastasis, beyond 5 cm of the primary tumor and multi-station lymph node metastasis, we will adopt IFI. The GTV with a 3.0–5.0 cm craniocaudal margin, a 0.6–0.8 cm lateral margin, and the GTV-N with a 1.0–1.5 cm margin, including the metastatic lymph nodes together make up the CTV (Figs. 2 and 3).

The PGTV will be 1.0 cm craniocaudally beyond the GTV-T and 0.5 cm radially and the GTV-N. Planning target volume (PTV) will be defined as 0.5 cm margin of the CTV for tumor motion and set-up variations. The typical contouring of the targeted tumors in different locations is depicted in Figs. 2 and 3.

SIB-IMRT will be given 5 days per week (i.e., Monday to Friday with the weekend off) for an average of 5.5 weeks. Radiotherapy will be delivered to achieve a prophylactic dosage of 50.4 Gy (1.8 Gy) to the PTV and 59.92 Gy (2.14 Gy) to the PGTV in 28 fractions. The contouring of the simulation images should include the lungs, heart, spinal cord, spinal cord planning OAR volume, and stomach on the CT scan. OARs such as the lungs, heart, spinal cord, and stomach will be delineated from their upper borders to their lower ends. The volume of lung tissue receiving 20 Gy or more should not exceed 28% of the total lung volume (i.e., V20 < 28%) and the V30 should not exceed 20%. The mean dose of the lung tissue should not be higher than 17 Gy (i.e., Dmean lung ≤17 Gy). Other dose constraints to the OARs include the following: V40 heart < 30%, V40 stomach < 40%, Dmean spinal cord = 9–21 Gy, and Dmax ≤45 Gy/6 weeks.

### Chemotherapy

The concurrent chemotherapy regimen consists of weekly doses of paclitaxel and a platinum-based drug. Paclitaxel will be given at a dose of 45–60 mg/m^2^, once a week, concurrent with radiotherapy for 5–6 weeks. The dose of the platinum-based drug (nedaplatin, lobaplatin, or cisplatin) is 20–25 mg/m^2^, once a week, concurrent with radiotherapy for 5–6 weeks. A total of 5–6 cycles of concurrent chemotherapy are recommended depending on the patients’ tolerance.

Consolidation chemotherapy within 1–3 months after the end of treatment will be recommended to appropriate and eligible patients who satisfy the following requirements: (1) Karnofsky performance status score ≥ 70 points, (2) ability to have semi-liquid or solid foods or receive nasal feeding, (3) no weight loss or loss of < 5% of the body weight, and (4) consent to undergo consolidation chemotherapy. The dose regimen for consolidation chemotherapy is as follows: paclitaxel 135–175 mg/m^2^ on day 1 and a platinum-based drug (nedaplatin, lobaplatin, or cisplatin) 50–80 mg/m^2^ on days 1–2 (lobaplatin 50 mg on day 1) every 3 weeks for 2–4 cycles starting 1–3 months after the completion of radiotherapy. Routine blood tests should be monitored every week, and hepatic and renal function should be checked during every chemotherapy cycle.

### Toxicity and adverse events

All treatment-related toxicities and adverse events will be graded with the RTOG toxicity criteria and the Common Terminology Criteria of Adverse Events (version 4.0). The detailed adverse events will be recorded in patients’ case report forms. Serious adverse events should be dealt with properly and reported to the institutional ethical review committee in 24 h, and the patients treated as promptly as possible. All patients with severe adverse reactions should be followed up until recovery.

Concurrent chemotherapy will be terminated, if ≥ grade 2 anemia, thrombocytopenia, or hepatic or renal dysfunction, ≥ grade 4 leukopenia/neutropenia, ≥ grade 3 radiation esophagitis, or other ≥ grade 3 non-hematological toxicities occur. If adverse events de-grade to grade 0–1 within 1 week of drug withdrawal, the patient can re-take chemotherapy as the required dose; otherwise, chemotherapy should be terminated. If ≥ grade 3 radiation pneumonitis occurs, both radiotherapy and chemotherapy should be terminated. The suitability of consolidation chemotherapy should be re-assessed within 4–8 weeks after radiotherapy, regardless of the grade of toxicities developed during definitive chemoradiotherapy.

### Statistical analysis and sample-size considerations

We assume that an estimated difference in 1-year OS of 33% (SIB arm) versus 50% (SIB + concurrent chemotherapy arm) [19] would justify applying this regimen in the future. Assuming a one-sided significance level of 0.05, a power of 0.80, and 10% of loss in each arm, a total of 164 patients (*n* = 82 in each group) would be needed in this trial. After using SAS software to generate a random number table, the patients will be randomly divided into two groups.

The rates of OS will be estimated using the Kaplan-Meier method, and the distributions of OS will be compared using the log-rank tests. Cox regression analysis will be used to identify prognostic factors for survival benefit.

### Ethics

The enrolled patients should be informed of the background of both treatment options, especially known efficiency and toxicities by the doctor-in-charge. It must be emphasized that both before and during the study, the patient is allowed to refuse the treatment. Before enrollment, the patients should sign literal informed consent. This study will be carried out accordance with the “Declaration of Helsinki” or the laws and regulations of the country under the supervision of the principal investigator, in order to provide the individual with greater protection. The institutional ethical review committee has approved with this study.

### Follow-up

Tumor regression should be assessed per the Response Evaluation Criteria in Solid Tumors (RECIST, version 1.1) within 1–2 months after the completion of treatment. The therapeutic effect on measurable metastatic lymph nodes and primary esophageal tumors will be evaluated using upper gastrointestinal contrast, endoscopy, EUS, neck/thoracic/upper abdominal enhanced CT/MRI, PET-CT (if necessary), etc.

The follow-up assessments will be done every 3 months for the first 2 year, every 6 months for 3–5 years, then every year. Routine follow-up assessments included: (a).

assessing tumor-related symptoms of dysphagia, chest tightness, hoarseness, cough, fever, etc., (b) laboratory investigations of blood routine examination, hepatic and renal function, tumor markers, etc., (c) image examinations of contrast-enhanced CT of the neck, thorax, and abdomen, ultrasonography of the neck and abdomen, upper gastrointestinal contrast, bone scan (if bone pain or abnormally elevated alkaline phosphatase), MRI of the brain (in case of any symptoms related to the central nervous system), etc., (d) recording of the patients’ vital signs, performance status, disease progression, subsequent treatment, nutrition, life quality, and any adverse events, etc.

### Quality assurance

A strict coordination and monitoring system will be constructed for this trial. First, a consist of physicians, dosimetrists, medical physicians, and research fellows’ team, named as Radiotherapy Trials Quality Assurance (RTTQA), has been created before the start of enrollment. A censor in charge of the RTTQA team will evaluate and audit the quality of data collected, communicate with the physicians from all participating centers.

In the assurance of treatment equality and quality of all involved centers, we have made great effort. We selected an EC case, an example by the RTTQA team, sent the case and CT imaging data to all participating centers at the start of the study. Then, all participating centers were requested to send the target delineation back to the RTTQA team. The RTTQA team assessed all collected cases for major and minor deviations. This is the first round of collection of target delineation (CTD). After that, a detailed protocol for target delineation was sent to the all centers and the physicians in charge contoured the targets again on the same sample case [20] and sent back again (second round of CTD). The RTTQA team examined the radiotherapy plans thoroughly and found both the quality and equality of the plans had improved significantly after two rounds of CTD. This procedure ensures that all centers and investigators have had the abilities and qualifications of planned test case before recruiting the patients. During the study, the censors from the RTTQA team will inspect randomly the quality of treatment, including images, target delineation, radiotherapy plans, and doses.

## Discussion

For unresectable EC, the National Comprehensive Cancer Network (NCCN) [21] and European Society for Medical Oncology (ESMO) [22] recommend a dose of 50–50.4 Gy for definitive radiotherapy with concurrent dual-drug intravenous chemotherapy (fluorouracil/capecitabine + a platinum-based drug), based on the RTOG 85–01 [3] and RTOG 94–05 studies [5]. However, these recommendations are based on 2DRT in the 1990s. The main cause of failure of this treatment is the high rate of locoregional recurrence (≥50%); moreover, treatment with a higher dose of 61–65 Gy with concurrent chemotherapy does not improve treatment outcomes as compared with the same regimen with a dose of 50 Gy [23]. Therefore, a reasonable radiotherapy dose supported by more research data is required. In the past several decades, few prospective studies have been conducted on the dose of 3DCRT, including SIB-IMRT, with concurrent chemotherapy. Retrospective analyses in our center show that for unresectable ECs, the 5-year OS (22.1–27.7%) after 3DCRT alone (median dose, 60 Gy) [14, 24] is higher than that after 2DRT (8.3–14.3%) [25]. These data confirm that the application of 3DCRT has improved the survival rate, and 3DCRT is now the main treatment for EC. Advancements in imaging technology have made radiotherapy more accurate, which may have improved its curative effects. Considering that radiotherapy (dose, > 50 Gy) with concurrent chemotherapy has been reported to yield 5-year OS rates of 26.0–44.3%, this treatment strategy is now the preferred option for EC [19, 26–28]. Compared with radiotherapy alone, radiotherapy with concurrent chemotherapy improves the 5-year OS rate by 2–11.1% [27, 29], which is different from the survival gap reported in RTOG85–01 (26% vs. 0%) [8]. Furthermore, 3DCRT is an effective treatment, second only to definitive chemoradiotherapy, especially for patients who cannot tolerate chemotherapy. Therefore, a prospective research study on this treatment strategy is required.

Currently, there is no international consensus on whether the draining lymph nodes need preventive irradiation in EC. A large body of data on three-field lymph node dissection in Japanese patients with EC has provided detailed lymph node metastasis sites and rates, and lends clinical support to the use of preventive regional lymph node irradiation in patients with unresectable EC (i.e., radiation to the high-risk lymph node metastasis area) [30, 31]. However, the dose required for preventive lymph node irradiation is different from that required for the primary tumor site. In the era of conventional radiotherapy technology, we had to undertake fractional or sequential treatments to meet the different dose requirement. However, by using reverse intensity modulation feature of IMRT, different radiation dose distributions can be administered to the nodal area and the primary tumor site at the same time. A phase II study of radical IMRT combined with concurrent chemotherapy for EC was performed with a similar dose as that used in the high-dose group of the RTOG 94–05 study. The median survival time (MST) was 23 months, and the 3-year OS rate was 44.4%, which indicates that SIB might be effective [32].

The use of SIB-IMRT is a novel aspect of our study. The long-term follow-up results of the RTOG 85–01 study showed that the major patterns of treatment failure were primary tumor persistence (radiotherapy: 37% vs. chemoradiotherapy: 25%) and locoregional failure (radiotherapy: 16% vs. chemoradiotherapy: 13%), which indicates that the local control rate for doses under 50.4 Gy is not satisfactory [33]. Thus, higher doses may be necessary for primary tumor areas, without increasing the toxicity to the surrounding normal tissue. One retrospective study also found that among ESCC patients, those who received high-dose irradiation (≥60 Gy) had better OS and local control rates than those who only received the conventional dose (50.4 Gy) [34]. Therefore, to explore this problem, we conducted a phase I/II radiation dose-escalation trial using the SIB technique with ENI and concurrent chemotherapy for unresectable EC [35]. We found that the SIB technique was feasible and safe at the maximum tolerated dose [95% PGTV/PTV = 59.92 (equivalent dose in 2-Gy fractions or EQD2 = 60.62 Gy)/50.40 Gy/28 f] concurrent with ENI and dual-drug chemotherapy for patients with unresectable EC. A total of 53 patients with SCC were enrolled in the above study. The median OS time, 1-year OS rate, and 1-year local failure-free survival were 31 months, 76.9, and 78.8%, respectively. Compared with a recent phase I/II trial of chemoradiotherapy with SIB radiotherapy for unresectable locally advanced EC (95% PGTV/PTV = 63.00 Gy/50.40 Gy/28 f, EQD2 = 64.31 Gy), our study had a better median OS, lower 1-year local recurrence rates, and similar 1-year OS and 1-year local recurrence rates (21.5 months, 30, and 78.3% respectively) [36]. However, all of these studies require long-term follow-up. Therefore, we intend to apply the above dose regimen in this phase III study to determine whether this regimen is safe, reliable, and promising.

The 5-year OS rate of EC patients has shown varying degrees of improvement after definitive radiotherapy with IMRT; even in the era of 2DRT, the 5-year OS was not 0%. Certain EC patients, such as those who are elderly or frail, those in poor health, and those with complications, are considered ineligible for esophagectomy. In such patients, definitive radiotherapy without major toxicity is considered a promising alternative. The NCCN and ESMO recommended dual-drug intravenous chemotherapy regimen (fluorouracil/capecitabine + a platinum-based drug) may cause severe acute and late adverse effects and is related to poor compliance rates in this specific population. Thus, radiotherapy alone might provide lower toxicity, and better survival and quality of life for these patients, and might be the preferred choice of treatment.

The widely accepted SIB-IMRT fractionated dose and total dose for preventive nodal irradiation are 1.8 Gy and 50.4 Gy, respectively. In contrast, the SIB-IMRT dose for the primary treatment area is controversial. The fractionated dose varies from 1.8 to 2.8 Gy; the total dose, from 62.5 Gy to 70 Gy; and the number of fractions, from 25 to 36, which reflects a wide variation [36–38]. Moreover, in the RTOG 85–01 and RTOG 94–05 studies, a total radiation dose of 64 Gy did not show significant benefits. Thus, a study to determine the appropriate radiotherapy dose and dose stratification is critical. Tan et al. reported that propensity score matching of 480 patients with ESCC receiving definitive radiotherapy or chemoradiotherapy (radiation dose: 50-70Gy) showed that: in 60-70Gy radiation dose range, there was no difference in OS rate between the radiotherapy group and chemoradiotherapy group (1, 3, and 5 years OS: 66.0, 35.6, 25.6% vs 63.6, 35.0, 25.3%, *p* = 0.833). While the OS rate after radiation and concurrent chemotherapy was significantly higher in the 50–59.9 Gy dose group (1, 3, and 5 years OS: 70.0, 36.4, and 32.3%; MST: 20 mouths] than in the radiotherapy group (1, 3, and 5 years OS: 57.1, 23.9, and 12.0%; MST: 15 months; *p* = 0.030) [24]. However, in the above study, the patients treated with this dose range (2.2–2.25 Gy/62.5–66 Gy/25–30 f) may be highly selected, for example, patients in whom the primary tumor was not sensitive to treatment, especially patients with EC who showed insignificant tumor regression during radiotherapy; or no signs of ulcer perforation without T4 stage. However, it is difficult to predict whether the tumor will be sensitive to radiotherapy before the treatment. Many studies on preoperative chemoradiotherapy/radiotherapy (neoadjuvant therapy) for EC have reported pathological complete response rates of 29–54.1% [39–44], while the rates of partial response or no response account for a higher proportion of patients. Moreover, pathological response is significantly associated with disease recurrence and survival [42–44]. In our phase I/II study, one EC patient received 2.17-Gy fractionated doses and 28-fraction radiotherapy, and he developed esophageal perforation during treatment. Therefore, whether SIB-IMRT (2.2–2.25 Gy/62.5–66 Gy/25–30 f) can replace conventional radiotherapy (1.8–2.0 Gy/50–50.4 Gy) as the standard treatment needs to be determined using phase III studies. A retrospective analysis of 2762 EC patients in China found that a total radiation dose of 60–61.9 Gy or 62–63.9 Gy in EQD2 produced the highest 5-year OS rates (31.7 and 34.7%, respectively); however, the 5-year OS rate was only 23–27.4% in the ≥64 Gy group [14]. Although survival is affected by various factors, this result indicates that more prospective studies are needed to find the reasonable dose. Establishing a reasonable total dose and fractionated dose is crucial for the clinical application of SIB-IMRT. However, there is no related evidence-based research to determine whether high-dose radiotherapy can yield better locoregional control and survival benefit for patients diagnosed with residual tumor during treatment.

Preventive regional irradiation and concurrent chemotherapy can improve the local control rate by eliminating micrometastases. However, whether these measures can increase the OS rate is not certain. It is reported that concurrent chemotherapy can increase the control of micrometastases, which might provide a possible survival benefit [33]. In the RTOG 85–01 study, the concurrent chemotherapy regimen consisted of cisplatin and fluorouracil. A 2012 randomized study of preoperative neoadjuvant chemoradiotherapy versus surgery alone for EC patients showed that the pathological complete response rate was 49% after weekly paclitaxel and carboplatin chemotherapy [45]. However, only 37 ESCC patients were recruited in this study. Thus, whether SIB-IMRT plus concurrent chemotherapy can be an alternative to conventional radiotherapy in ESCC patients’ needs to be determined.

In this paper, we propose a prospective, multicenter phase III clinical trial to obtain high-level type I evidence for a safe and effective therapeutic regimen for patients with unresectable EC. We will compare SIB-IMRT with or without concurrent paclitaxel + nedaplatin chemotherapy with the addition of consolidation chemotherapy for advanced EC.

## Data Availability

Not applicable – data collection is still ongoing.

## Abbreviations

IMRT: Intensity-Modulated Radiotherapy
EC: Esophageal Cancer
SIB: Simultaneous Integrated Boost
EGJC: Esophagogastric Junction Cancer
RTOG: Radiation Therapy Oncology Group
OS: Overall Survival
2DRT: Two-Dimensional Radiotherapy
3DCRT: Three-Dimensional Conformal Radiotherapy
OAR: Organs at Risk
PTV: Planning Target Volume
PGTV: Planning Gross Tumor Volume
UNL: Upper Normal Limit
CT: Computed Tomography
GTV-T: Gross Tumor Volume
EUS: Endoscopic Ultrasonography
PET-CT: Positron-Emission Tomography
GTV-N: Metastatic Regional Nodes
CTV: Clinical Target Volume
ENI: Elective Nodal Irradiation
IFI: Involved-Field Irradiation
RECIST: Response Evaluation Criteria in Solid Tumors
RTTQA: Radiotherapy Trials Quality Assurance
CTD: collection of target delineation
NCCN: National Comprehensive Cancer Network
ESMO: European Society for Medical Oncology
MST: Median Survival Time
ESCC: Esophageal Squamous Cell Carcinoma
EQD2: Equivalent dose in 2-Gy fractions
